# *Ubiquitin A-52 residue ribosomal protein fusion product 1* (*Uba52*) is essential for preimplantation embryo development

**DOI:** 10.1242/bio.035717

**Published:** 2018-08-22

**Authors:** Jiude Mao, Chad O'Gorman, Miriam Sutovsky, Michal Zigo, Kevin D. Wells, Peter Sutovsky

**Affiliations:** 1 Division of Animal Sciences, University of Missouri, Columbia, MO 65211, USA; 2 Department of Obstetrics, Gynecology and Women's Health, University of Missouri, Columbia, MO 65211, USA

**Keywords:** Uba52, Development, Embryo, Ribosomal, Ubiquitin

## Abstract

*Ubiquitin A-52 residue ribosomal protein fusion product 1* (*Uba52*), a ubiquitin-ribosomal fusion gene, is a major source of ubiquitin protein for covalent modification of proteinaceous substrates recycled by ubiquitin-proteasome system (UPS). Its role in early embryo development has not been studied. Using the CRISPR/Cas9 gene editing tool, the objective of this study was to determine if UBA52 protein is required for mammalian embryogenesis. Matured metaphase II porcine oocytes were injected with CRISPR Cas9+guide RNAs (Uba52 gRNA) or Cas9 without gRNAs as control, followed by *in vitro* fertilization (IVF) and embryo culture to day 7. Injection of Cas9+gRNAs affected embryo development. On day 4 of embryo culture, the proportion of 2-, 4- and 8-cell stage embryos was significantly different between the Uba52 gRNA and control group (*P*<0.05), with more 8-cell stage embryos in the control and more 4- and 2-cell stage embryos in the Uba52g RNA group. This delay in the development of Uba52 gRNA embryos occurred at the transition from the 4- to 8-cell stages, around the time of major zygotic genomic activation. The percentage of blastocyst formation on day 7 and the cell number per blastocyst were significantly lower in the Uba52 gRNA group than in the control (*P*<0.05). Genotyping by PCR and DNA gel electrophoresis analysis showed that 91.8% of embryos that failed to develop to blastocyst had either a monoallelic or a biallelic modification of the *Uba52* gene. In comparison, only 24.4% of embryos that reached blastocyst had a monoallelic modification and biallelic editing was not found in any of the blastocysts. Based on immuno-labeling intensity, both UBA52 and proteasome protein levels on days 4 and 7 of culture were significantly lower in the Uba52 gRNA group than in the control (*P*<0.05), in agreement with UBA52 western blotting-densitometry of day 4 embryos. Morphological examination of blastomere nuclei revealed abnormal nuclear structure in the Uba52 gRNA group, such as reduced size, irregular shapes, nucleus fragmentation and uneven DNA distribution at all stages of embryo development. Nuclear morphology studies of embryos injected with Cas9+gRNAs and co-injected with plasmid DNA encoding nuclear localized GFP further supported these observations. In conclusion, our data indicate that the *Uba52* gene is essential in early embryogenesis.

## Abstract (precis)

**Summary:** *Ubiquitin A-52 residue ribosomal protein fusion product 1* (*Uba52*), a ubiquitin-ribosomal fusion gene, is a major source of ubiquitin protein for covalent modification of proteinaceous substrates recycled by ubiquitin-proteasome system. Using CRISPR/cas9 gene editing tool, we demonstrate that that Uba52 gene is essential in early embryogenesis in mammals.

## INTRODUCTION

Ubiquitin (UBB/UBC/UBD) is a 76-amino acid small protein with molecular mass of 8.5 kDa. It is a highly conserved protein in eukaryotes, with a fundamental role in selective protein degradation by the ubiquitin–proteasome system (UPS). In this pathway, ubiquitin molecules are attached covalently to the substrate proteins, a process called ubiquitination, and mediated by a multi-enzymatic complex including ubiquitin-activating enzymes E1 (UBA1), ubiquitin-conjugating enzymes E2 (e.g. UBE2A, UBE2B, UBE2C), ubiquitin ligases E3 (e.g. UBE3A and others), and ubiquitin chain elongation/ubiquitination factors enzymes E4 (e.g. UBE4A/UBE4B), which work sequentially in a cascade (Sutovsky, 2003). Ubiquitinated substrates are most commonly degraded by the 26S proteasome (Ciechanover and Schwartz, 1994). Besides cellular homeostasis/protein turnover, the UPS has been implicated in the pathogenesis of many diseases (Hershko and Ciechanover, 1998), as well as in the physiological events of mammalian fertilization and embryogenesis (Sutovsky, 2003), sperm function (Sutovsky et al., 2001) and the control of mitochondria inheritance (Song et al., 2016). In addition to its role in protein degradation, the non-proteolytic consequences of protein ubiquitination also play an important role in cellular functions such as signaling, cell cycle control, transcriptional regulation and apoptosis (Komander and Rape, 2012).

Ubiquitin is encoded by multiple genes including monomeric UB-ribosomal fusion genes, *Uba52* and *RPS27A*, that encode one UB unit fused to a ribosomal protein, and polyubiquitin genes, *UBB*, *UBC* and *UBD* that harbor up to ten tandem repeats of monoubiquitin coding units (Baker and Board, 1991; Finley et al., 1989; Wiborg et al., 1985). While the polyubiquitin genes play a key role in stress-responses such as heat shock, starvation (Finley et al., 1987; Fornace et al., 1989), DNA damage, oxidative stress (Vihervaara et al., 2013) and heavy metal cytotoxicity response (Lee et al., 2015), the ubiquitin-ribosomal fusion genes are stably expressed to satisfy the demand for ubiquitin under basal conditions (Bianchi et al., 2015). The *Uba52* gene has been identified as a housekeeping gene (Warrington et al., 2000) and the transcript has been used as a reference in rhesus monkey (Ahn et al., 2008), starfish (Sadritdinova et al., 2014) and bovine tissues (Schoen et al., 2015), to quantify gene expression. Through cDNA microarray analysis of gene expression in porcine oocytes and early preimplantation embryos, *Uba52* expression in the blastocyst stage embryo was six times higher than the metaphase II oocytes (Whitworth et al., 2005). A similar expression pattern was observed in rhesus monkey (Mtango and Latham, 2007) and mouse (Zeng et al., 2004) embryos. This suggests that *Uba52* has a functional role in early embryogenesis. Indeed, the *Uba52* and other UPS gene expression was dysregulated in the aberrant rhesus monkey embryos with reduced developmental potential (Mtango and Latham, 2007). In spite of gene expression profiles, there have been no studies to determine its roles in early embryogenesis. We thus hypothesized that this ubiquitin fusion protein was essential in mammalian embryo development.

To study the specific roles of a gene, gene mutation approaches have been used. Recently, the bacterial clustered regularly interspaced short palindromic repeat (CRISPR)/CRISPR-associated system (Cas9), has become increasingly popular for creating gene edits in both the somatic cells and embryos to study gene function (Cho et al., 2013; Cong et al., 2013). The CRISPR/Cas9 system has been used to efficiently generate genetically modified animals via zygotic injections of Cas9 and guide RNA (gRNA) in many species, such as the mouse (Wang et al., 2013), pig (Hai et al., 2014; Whitworth et al., 2017) and monkey (Niu et al., 2014), indicating its versatility and universality. High specificity and efficiency of gene editing as well as low cost and ease of application has helped to promote its widespread use in biomedical research, including the increasingly important domestic pig model. Moreover, injecting multiple CRISPR guides at the same time can increase the possibility of gene editing (Spate et al., 2016).

The purpose of this study was to determine if *Uba52* gene expression is required for early embryogenesis by injecting Cas9 and guide RNAs into metaphase II arrested porcine oocytes, and examining embryo development up to and including the blastocyst stage. To our knowledge, this is the first report that *Uba52* gene modification/knockout causes development lethal arrest prior to embryo implantation, indicating its importance in mammalian embryogenesis.

## RESULTS

### Comparison of embryo development between the Uba52 gRNA and control group

**Fig. 1.**
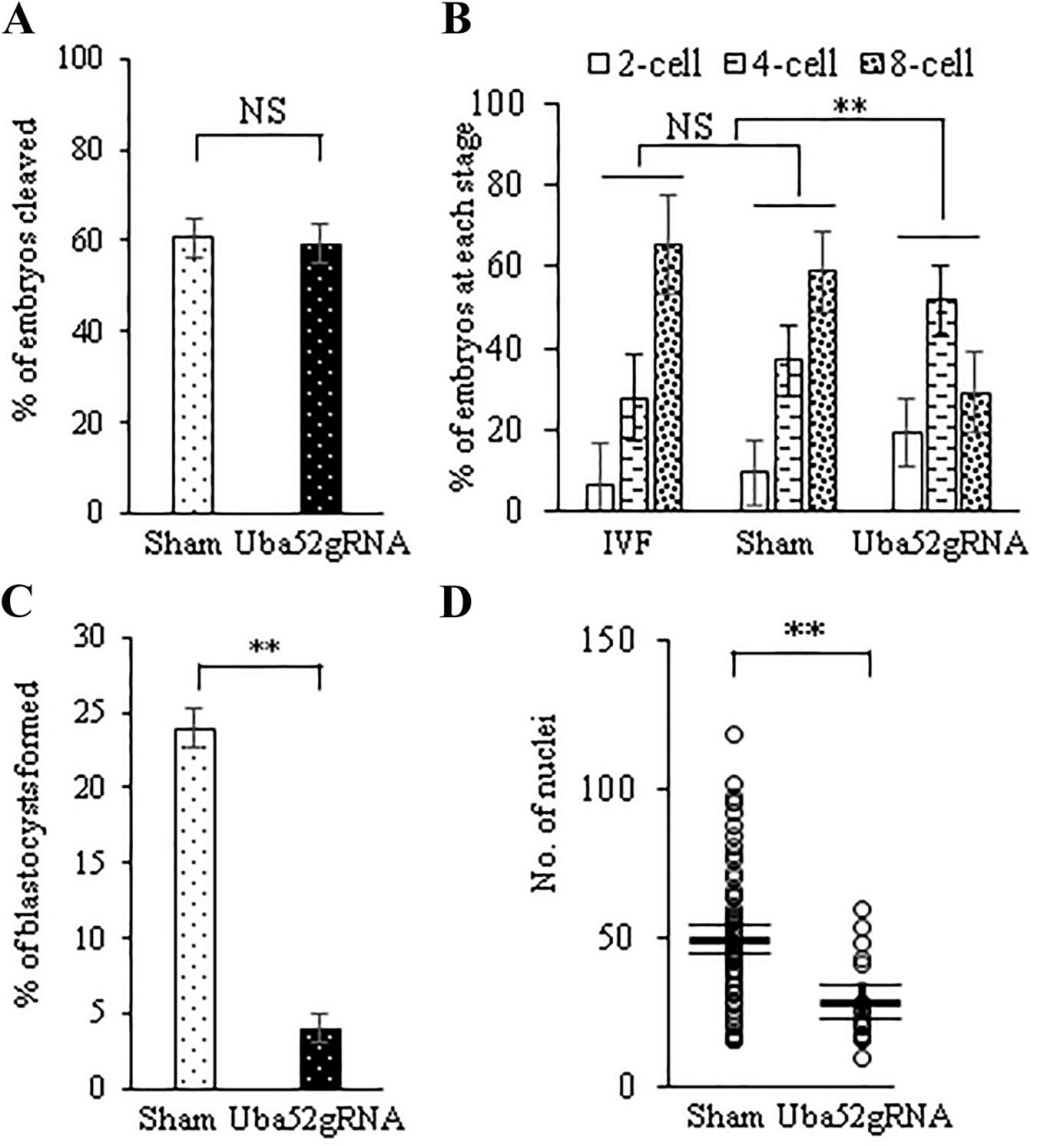
**Embryo development on days** **2, 4 and 7 of culture.** (A) Per cent of embryos cleaved on day 2 of culture (Day of IVF=0; *n*=784 for the control group and 1150 for the Uba52 gRNA, in 12 replicates). (B) Distribution of 2-, 4- and 8-cell stage embryos on day 4 in the non-manipulated IVF embryos (*n*=86 from three replicates), manipulated control and Uba52gRNA group (same embryos from day 2, as in A). The distribution of embryos at each stage was not different between IVF and sham groups. The Uba52 gRNA group was significantly different from both IVF and sham control by X^2^ test. (C) Blastocyst formation at day 7. Per cent of blastocysts formed in the Uba52gRNA group was lower than that in the sham control (*P*<0.01). (D) Cell number per blastocyst from 70 control and 17 Uba52 gRNA blastocysts of three replicates. NS, not significant; ** indicates difference between the Uba52gRNA and control groups at *P*<0.01.

The objective was to compare the developmental potential of control and gene-edited embryos. There were two groups: the Uba52 gRNA group injected with CRISPR/Cas9+gRNAs and the sham control group injected with CRISPR/Cas9 without gRNAs. A total of 1934 injected oocytes (control: 784; Uba52 gRNA: 1150) from twelve replicates were used for embryo development study. The percentage of embryos cleaved on day 2 (Fig. 1A) was not different between the Uba52 gRNA group and control (59.2±4.2 versus 60.6±4.2%, respectively). However, on day 4 of culture, the distribution of embryonic developmental stages was different between the two groups. Compared to the sham control, the Uba52 gRNA group had more embryos at 2-cell (19.2±8.2 versus 9.4±8.2%) and 4-cell stage (51.8±8.5 versus 36.9±8.5%), and fewer reaching the 8-cell stage (29.0±9.9% versus 58.7±9.9; *P*<0.05). The micro-manipulated control group without gRNA was not different from the non-manipulated *in vitro* fertilization (IVF) control, and both groups were different from the Uba52 gRNA group (Fig. 1B). Thus, the delay in the development of Uba52 gRNA embryos to 8-cell stage was accrued at the transition from the 4-cell to 8-cell stage, which in the domestic pig coincides with the degradation/depletion of maternally stored proteins, and major zygotic genome activation.

Blastocysts were morphologically evaluated on day 7. Embryos that had cavitated and possessed a normal or thinning zona pellucida, were considered to be blastocysts. The developmental arrest observed on day 4 in the Uba52 gRNA group resulted in a significantly lower percentage of blastocyst formation compared to the sham injected controls (4.0±1.0 versus 23.9±1.3%, *P*<0.01) on day 7 (Fig. 1C). In addition, the average number of nuclei in the Uba52 gRNA embryos that did develop to blastocyst on day 7 (Fig. 1D) was lower than that in the control group (28.8±6.5 and 49.2±2.8 for the Uba52 gRNA and control group, respectively; *P*<0.01).

### Genotyping of *Uba52* gene in day 7 blastocysts and arrested embryos

**Fig. 2.**
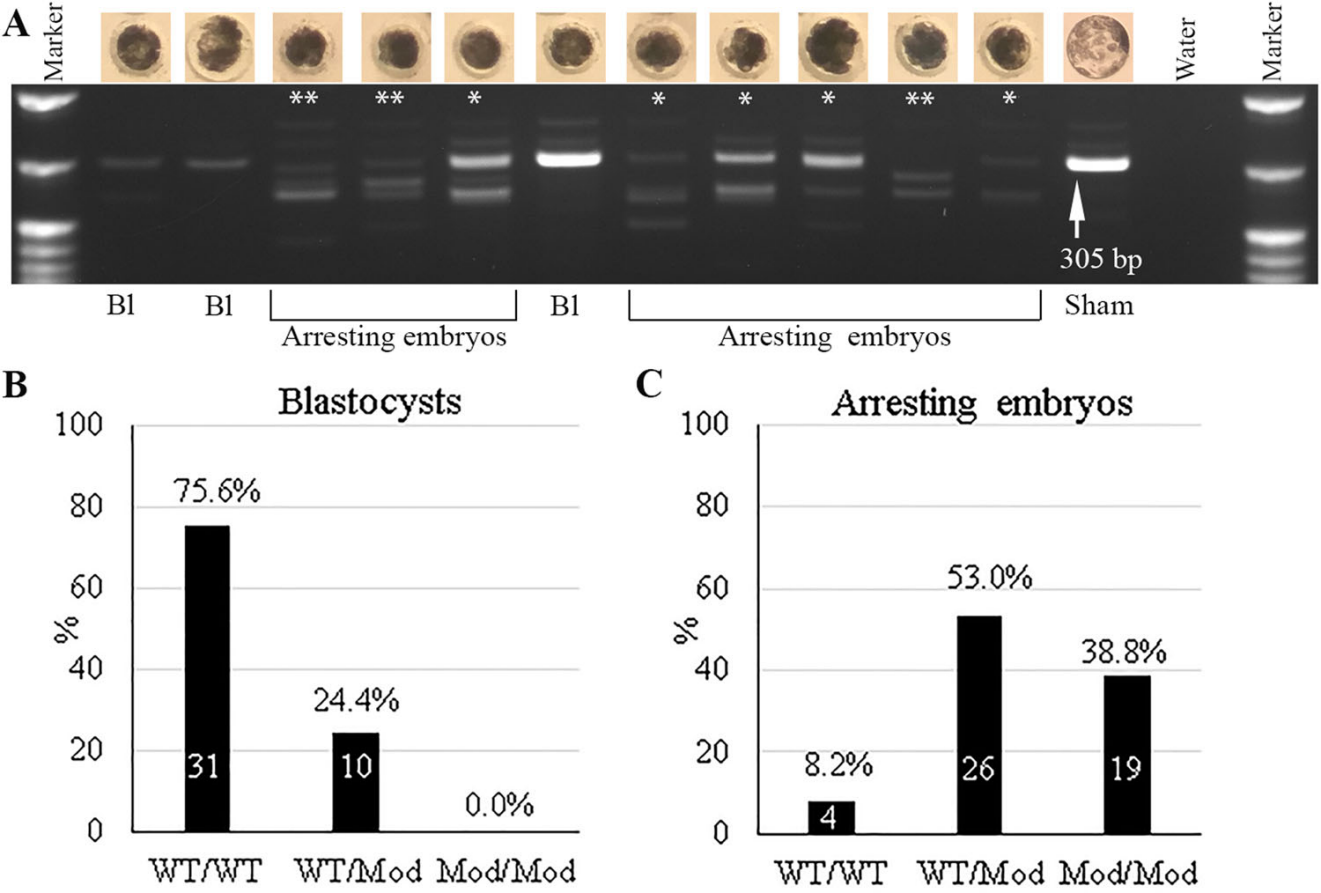
**Genotyping of Uba52 gene in day 7 blastocysts and arrested embryos.** Genotyping of *Uba52* gene by DNA gel electrophoreses (A), distributions of WT/WT, WT/Mod, and Mod/Mod genotypes in the day 7 Uba52 gRNA blastocysts (B) and arrested embryos (C). Bl, blastocyst. * and ** indicate monoallelic (WT/Mod) and biallelic (Mod/Mod) modification of *Uba52* gene, respectively. Control and manipulated WT/WT blastocysts had a single band at 305 bp (arrow in A).

To determine the *Uba52* gene modifications in early embryos, a total of 90 day 7 embryos, including 41 blastocysts and 49 arrested 4-cell- to morula-stage embryos were assayed by PCR and gel electrophoresis (Fig. 2A,B). Of the 41 blastocysts, thirty-one embryos (75.6%) only had wild-type (WT) bands and classified as WT/WT. Ten blastocysts (24.4%) had one WT band and one lower band (deletion), signifying monoallelic modification (WT/Mod). No biallelic modification of *Uba52* gene (classified as Mod/Mod) was found in day 7 blastocysts. To confirm that there was no *Uba52* gene modifications in the wild-type bands of day 7 Uba52 gRNA embryos, four WT/WT blastocysts were further Topo cloned and *Uba52* gene was sequenced. There was no modification of *Uba52* gene detected in such blastocysts, confirming the genotyping results of Uba52 by PCR and gel electrophoresis.

**Fig. 3.**
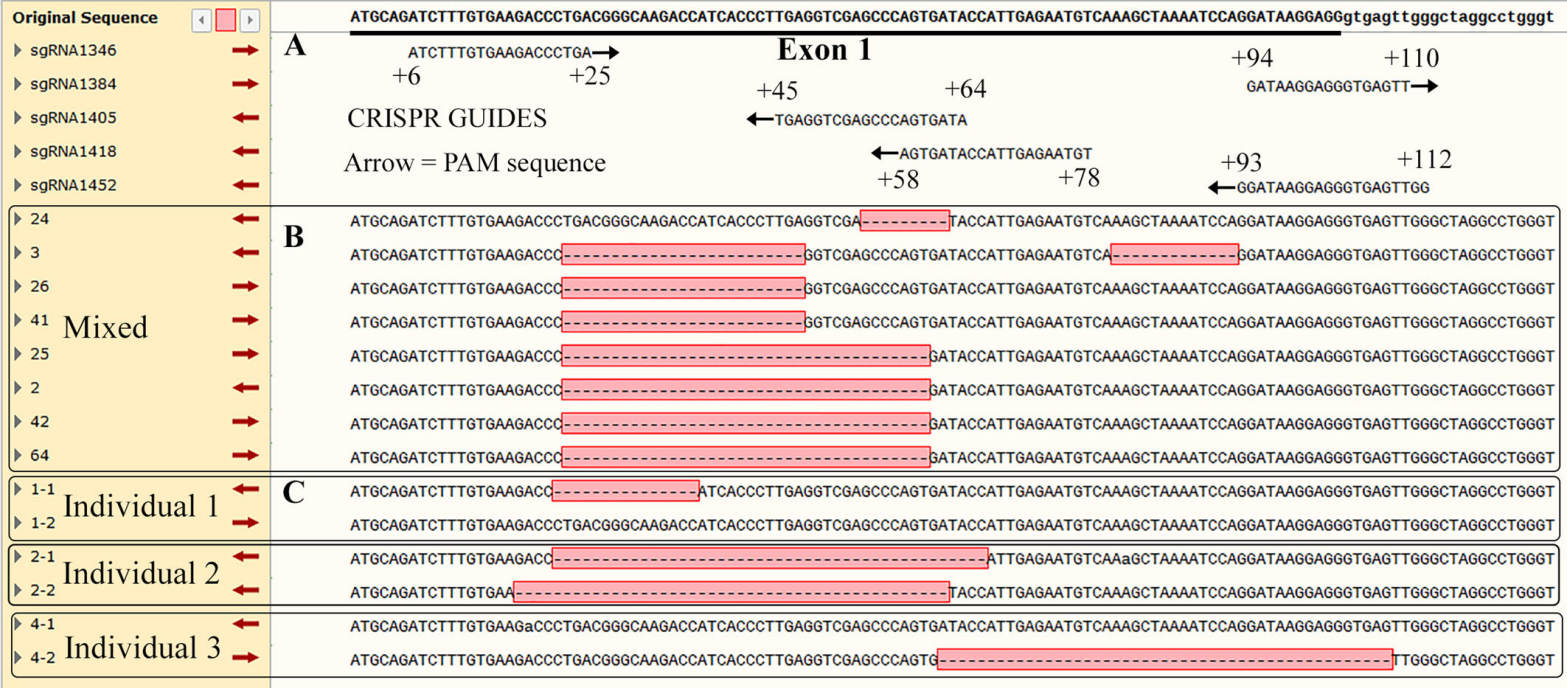
**Topo cloning and sequencing of *Uba52* gene in the modified embryos.** (A) Location of guides flanking exon 1 of the *Uba52* gene and potential cutting sites indicated by PAM (protospacer adjacent motif). (B) Sequencing of six pooled modified embryos to show cutting sites. (C). Sequencing of individual embryos to confirm monoallelic (individuals 1 and 3) and biallelic (individual 2) modifications.

Among the 49 arrested day 7 embryos, four embryos showed only wild type (8.2%), 45 embryos (91.8%) had either monoallelic (26 embryos, 53.0%; indicated by one star in Fig. 2A) or biallelic modification (19 embryos, 38.8%; indicated by two stars in Fig. 2A). Again, the *Uba52* modified embryos identified by DNA gel electrophoresis were sequenced and confirmed to be *Uba52* modification (Fig. 3). These data suggest that one allele of the *Uba52* gene was sufficient to support development to blastocyst in some embryos, but biallelic modified embryos could not develop. Thus, *Uba52* expression appears to be essential for embryo development.

### Decreased UBA52 and proteasome subunit content in the Uba52 gRNA embryos

**Fig. 4.**
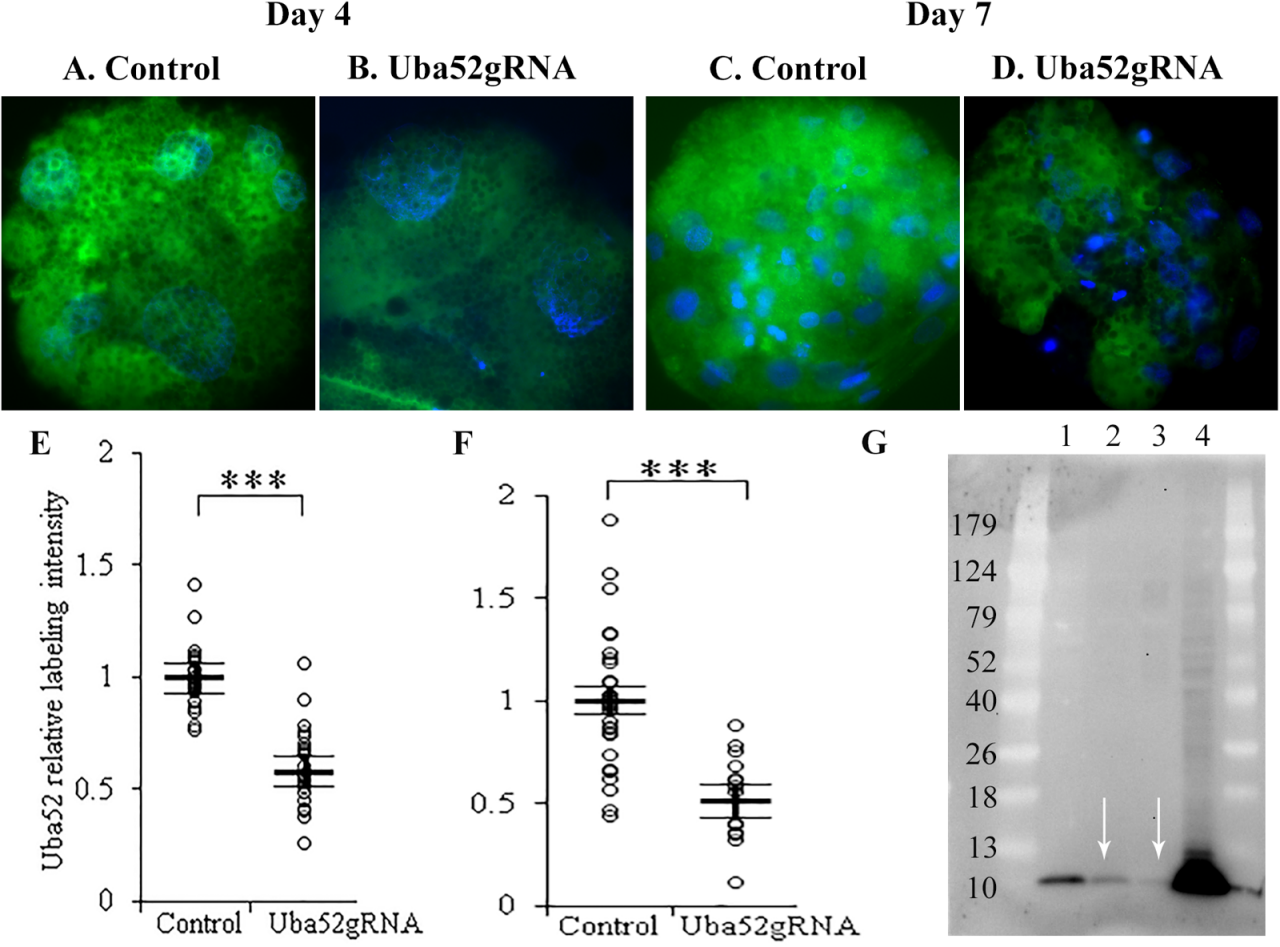
**Immunoreactive UBA52 protein in day** **4 and day 7** **embryos.** (A-D) Representative images of day 4 and 7 embryos immuno-labeled for UBA52 (green) and DNA (blue). (E,F) Quantification of fluorescence intensity of UBA52 on day 4 (E) and 7 (F). Relative intensity values were adjusted so the average of the control group was equal to 1. Fluorescence intensity of UBA52 was reduced by the Cas9+gRNA injection (***P<0.001). Bar lines are LS-means±s.e.m. (G) Western blotting of day 4 embryos confirmed the reduction of UBA52 band in Cas9+gRNA injected embryos (lane 3 versus 2). Lanes 1, 2, 3 and 4 represent 62 MII oocytes, 45 control embryos, 45 Uba52 gRNA embryos and a total of 100,000 unspecified fibroblast cells. Arrows indicate predicted Uba52 bands.

The objective of this experiment was to evaluate the UBA52 and proteasomal subunit protein content in the control and Uba52 gRNA embryos on day 4 and 7 of culture by immunostaining. Both *Uba52* and proteasomal subunit gene expression changes have been associated with developmental potential of mammalian oocytes and embryos (Mtango and Latham, 2007). UBA52 protein was localized in both the cytoplasm and nuclei of embryo blastomeres (Fig. 4A-D). The intensity of immune-reactive UBA52 in the Uba52 gRNA group was significantly lower on days 4 and 7, compared with control (Fig. 4E,F; *P*<0.01). To confirm the specificity of UBA52 immuno-labeling, western blotting (WB) was carried out on day 4 embryos to determine their UBA52 protein content. The density of UBA52 band, which migrated consistently at the predicted size of ∼14.5 kDa, was lower in the Uba52gRNA group than in sham control (band density was normalized against residual protein load, with background subtraction: 25.5 versus 12.0, arbitrary units, for the sham and Uba52gRNA groups, respectively; lanes 2 and 3 in Fig. 4G), which agrees with immuno-labeling analysis.

**Fig. 5.**
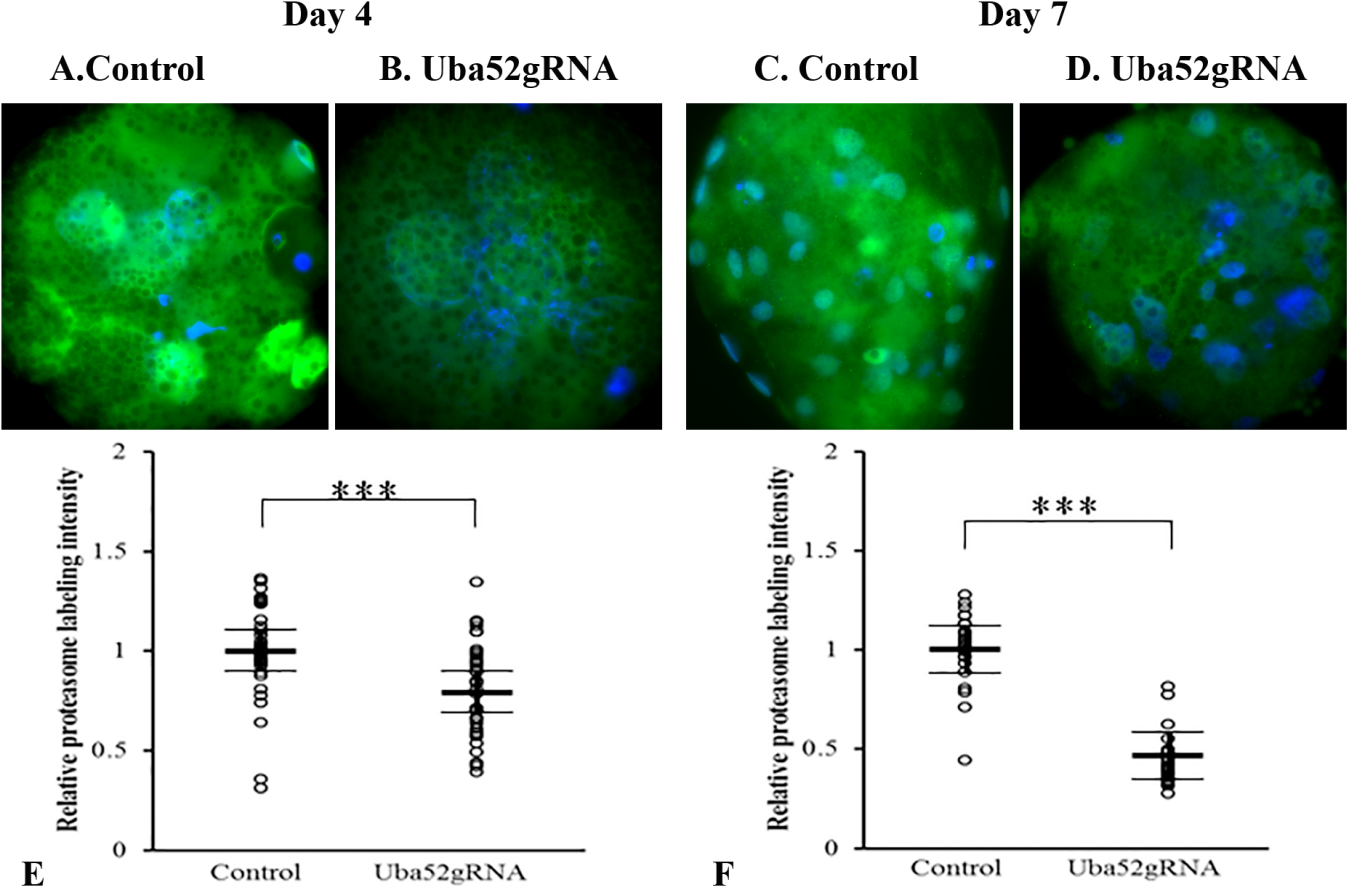
**Quantification of proteasomal subunit proteins in the day** **4 and day** **7 embryos.** (A-D) Representative images of embryos that were immuno-labeled for proteasome (green) and counter stained for DNA (blue). (E-F) Scatter plots of proteasome labeling intensity for day 4 (E) and day 7 (F) embryos. *** shows that immunoreactive proteasome was lower in the Cas9+gRNAs group than the control (*P*<0.001). Relative intensity values were corrected so the average of the control group was equal to 1. Bar lines represent LS-means±s.e.m.

Being previously tied to developmental potential/competence of mammalian oocytes and embryos, the localization (both nuclear and cytoplasmic) and relative abundance of proteasomal subunit proteins was evaluated with immunocytochemistry with a previously characterized antibody (Sutovsky et al., 2004; Zigo et al., 2018) recognizing multiple 20S proteasomal core subunits (Fig. 5). As would be expected, the labeling intensity of proteasomes was stronger inside the nucleus than the cytoplasm (Fig. 5A-D). The combined nuclear and cytoplasmic intensity of immune-reactive proteasomes in the Uba52 gRNA group was significantly lower (*P*<0.01) than control, both on day 4 and day 7 (Fig. 5E,F), with a reduction by 20% on day 4 and more than 50% on day 7 in the Uba52 gRNA versus control.

Taken together, these experiments showed that CRISPR/Cas9+Uba52 gRNAs injection efficiently lowered the content of UBA52 protein and proteasomal subunits in the embryos, and disrupted embryo development on both day 4 and day 7.

### Nuclear morphology abnormalities in the Uba52g RNA embryos

**Fig. 6.**
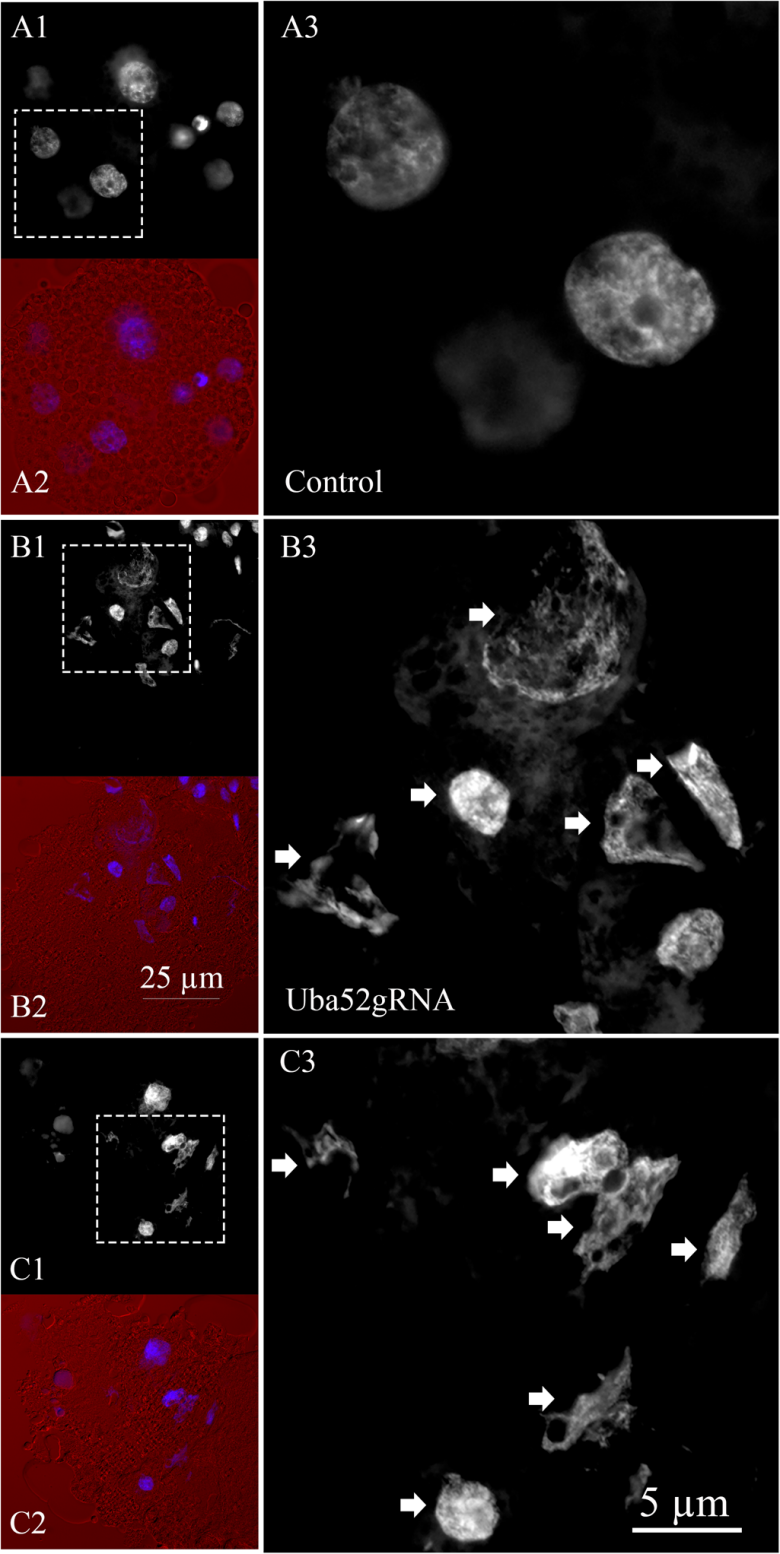
**Nuclear morphology of day** **4 embryo stained with Hoechst 33342.** (A) A control 8-cell embryo. (B,C) Representative Uba52 gRNA embryos. Grayscale panels show DNA staining, pseudo-colored panels show overlay of DNA (blue) and bright field (red) images. A3, B3 and C3 are four times magnified rectangles traced in panels A1, B1 and C1. Compared to control, all nuclei in the Uba52 gRNA embryos show morphological abnormalities, indicated by the arrows.

To understand the importance of the *Uba52* gene and protein for the maintenance of nuclear structure, the morphology of blastomere nuclei was examined in live 4- and 8-cell stage embryos (day 4) after DNA staining with Hoechst 33342. Invariably, the blastomere nuclei of 42 sham-injected embryos had a regular, oval shape (Fig. 6A). In contrast to the control embryos, the Uba52 gRNA embryos (a total of 55 from three replicates) displayed various morphological abnormalities, ranging from large sized (compared to control), oval-shaped nuclei with densely aggregated chromatin and uneven DNA distribution, to small, disorganized and irregularly shaped nuclei (arrows in Fig. 6B,C). The small nuclear size in the Uba52 gRNA embryos could be either the results of degeneration and fragmentation of previously regular blastomere nuclei or it could originate from single, detached mitotic chromosomes making karyomeres/micronuclei after entering interphase.

### GFP coinjection with Cas9+gRNAs confirmed nuclear morphological abnormalities

**Fig. 7.**
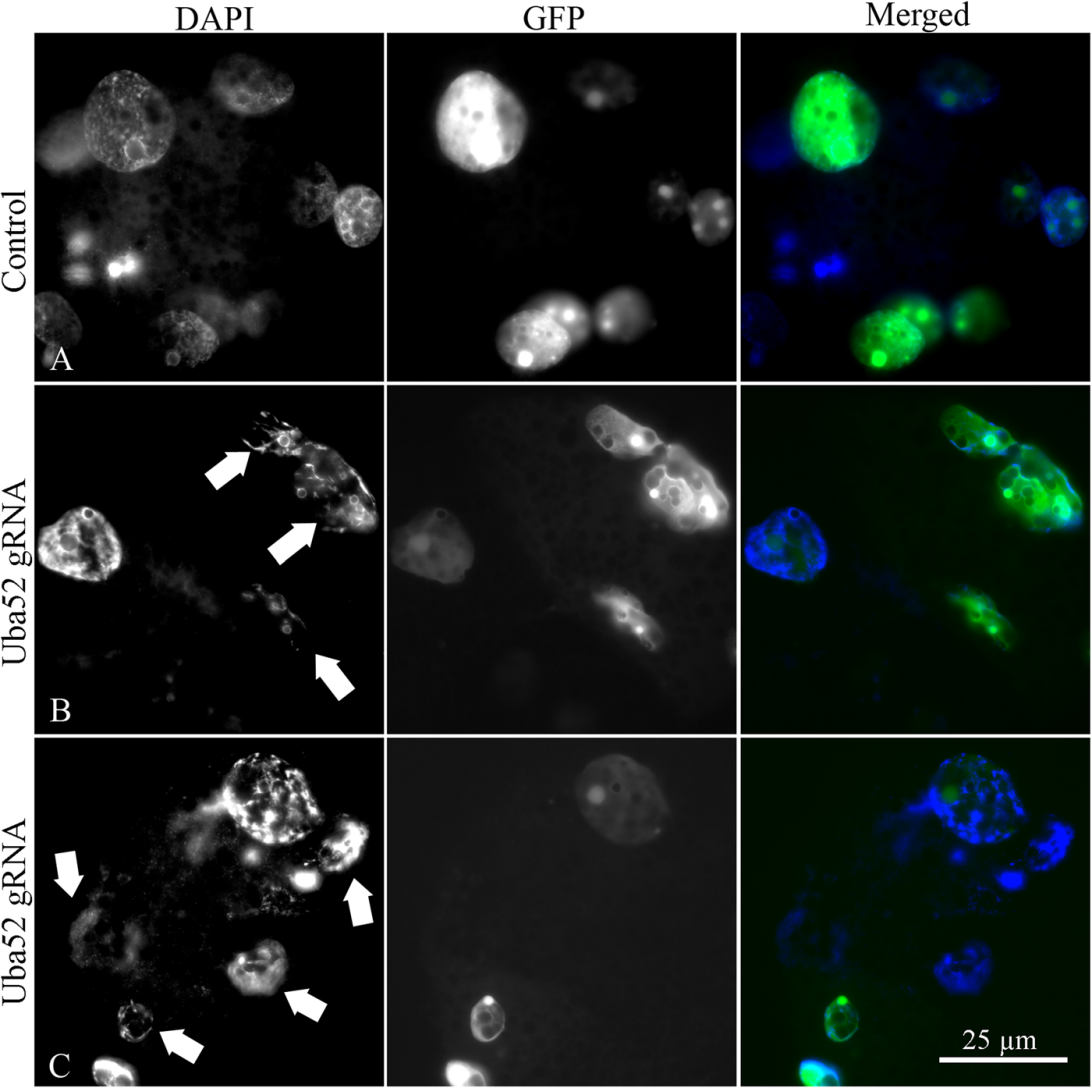
**Representative images of control (Cas9+GFP) and Cas9+gRNA+GFP co-injected embryos on day** **4 of culture, showing nuclear-imported GFP (green) and DNA counter-staining with DAPI (blue).** (A) 8-cell stage control embryo. (B,C) Cas9+gRNAs+GFP injected embryos showing abnormal nuclear morphology (arrows) at 6- and 8-cell stage, respectively.

The green fluorescent protein (GFP) is often used in many studies to determine the subcellular localization of other proteins by analyzing fusion proteins. A similar approach to examine the nuclear morphology of embryo blastomeres was used by injecting Cas9+gRNAS with plasmid DNA encoding nuclear-import signal GFP construct before fertilization. The embryos were collected on day 4 of culture, fixed, and stained with DNA stain DAPI. The nuclei of control group (Cas9+GFP without gRNAs) were morphologically normal, with regular size and oval shape (Fig. 7A). In the Uba52gRNA group, various blastomere-nuclear abnormalities were observed at the developmental stages ranging from 2-cell to 8-cell (Fig. 7B,C), including irregular shapes, smaller size and uneven hyper-condensed chromatin. Lower intensity of the nuclear GFP fluorescence was noticeable in the 8-cell Uba52gRNA embryos, compared to 8-cell control, and 2- to 6-cell control and Uba52gRNA embryos. The nuclear perimeter of in the Uba52 gRNA embryos was often uneven, resulting in a patched appearance of GFP. These results confirm that *Uba52* gene modification resulted in embryonic nuclear abnormalities, which may have contributed to the observed developmental arrest.

## DISCUSSION

Modification of *Uba52* gene by CRISPR/Cas9 technology had a dramatic effect on the development of porcine embryos *in vitro.* There was a significant reduction in the number of blastocysts formed on day 7 of embryo culture and the genotyping of the genetically edited embryos injected with Uba52 gRNA confirmed that over 91% of them carried monoallelic or biallelic modifications of the *Uba52* gene. Furthermore, the blastomere nuclei of the Uba52 gRNA embryos displayed abnormal nuclear morphology and highly variable size, often being much smaller than the sham control nuclei. Proper timing of key developmental processes during the early preimplantation period is very important for the attainment of blastocyst stage. The genome-wide sweep of maternal mRNAs and proteins, DNA replication, chromatin remodeling and major zygotic genome all have to be precisely coordinated. Many of these early processes are actually regulated by UPS (Gilberto and Peter, 2017; Moreno and Gambus, 2015). The timely onset of the expression of key cell cycle regulators is essential for normal development, and aberrations can lead to apoptosis and developmental arrest (Hara et al., 2006). Correct expression of cell cycle regulators during preimplantation development is critical in order to sustain developing embryo response to DNA damage, stress and other adverse conditions by activating either survival and repair mechanisms, or apoptotic processes. Modification of *Uba52* gene in the present study likely represents a multipronged insult to several key functions of the blastomere, resulting in a lethal developmental arrest. Accordingly, mutation of *Uba52* in mice is embryo lethal, affecting embryo ubiquitin levels, ribosome assembly, cell cycle progression and overall protein synthesis (Kobayashi et al., 2016). While the *Uba52* deficient mouse fetuses die before embryonic day 10.5, it is possible that they develop to blastocyst and implant. In contrast, porcine zygotes in the present study became arrested as early as the 4-8 cell stage of preimplantation development and no blastocyst with biallelic *Uba52* modification were found. This discrepancy could be due to species difference but also to compensate for related ubiquitin-ribosomal fusion protein genes which may occur when the gene is deleted completely, but not when a truncated dysfunctional protein is being produced due to a gene modification (present study). Mutation of *Uba52* homologs in the *Saccharomyces cerevisiae* showed that the UBI1 and UBI2 double mutant is not viable (Finley et al., 1989); UBI1 and UBI2 genes in yeast encode identical (at amino acid sequence level) ubiquitin-tail ribosomal protein.

Ubiquitin is the most abundant protein in the Eukaryotic cells, representing up to 5% of total protein. However, the free unconjugated ubiquitin pool is surprisingly small, which means that, despite its pervasive roles in many cell functions, ubiquitin is not produced in excess. The embryonic genome becomes activated at 4-cell stage in the pig. During normal development from zygote to 4-cell stage embryo, transcripts involved in protein catabolic processes are highly abundant at both the 2- and 4-cell stages. Many of them are directly linked to ubiquitin (Østrup et al., 2013). However, when *Uba52* gene was modified mono- or bi-allelically in the present study, residual ubiquitin and maternal Uba52 mRNA pools may have been sufficient to support embryo development for a short time, enabling cell cycle progressing, mitosis, RNA translation and processing (including ribosomal machinery), protein catabolism and chromatin remodeling (Østrup et al., 2013). Thus, the gene modified embryos still could cleave and develop to 4-cell and even to 8-cell stage as we observed. However, after 4 days of culture, there was a statistically significant reduction of the UBA52 protein content in the Uba52 gRNA group compared to control. Such a reduction would represent a major insult to many critical cell functions, and result in a development delay leading to a failure of blastocyst formation in the biallelically modified embryos. Since *Uba52* is a housekeeping gene, it may have to be activated along with other housekeeping genes in advance of the major genome activation to support essential cellular functions (Latham and Schultz, 2001). As UBA52 protein contributes both to free ubiquitin pool and to ribosomal protein complex, it is reasonable to assume that many cell key functions such as cell cycle control and sustaining nuclear structures could have been impaired by UBA52 deficiency. In the present study, the majority of embryos arrested at 8-cell stage, i.e. within one cell cycle after the zygotic genome activation, indicating that the genome activation itself may also be affected. The observed reduction of embryo proteasome content would further aggravate the effect of *Uba52* obliteration on protein synthesis and turnover.

As demonstrated in normal cell division (Amin et al., 2008), major changes in nuclear structure are required to allow segregation of duplicated chromosomes to the daughter cells during mitosis. However, for the porcine embryo, it takes over 12 h to complete each cell cycle between fertilization and the 8-cell stage (Mateusen et al., 2005). The nuclei in all the control embryos had evenly distributed DNA/heterochromatin. In the early control embryo blastomeres, the complex spatial arrangements in the nucleus are maintained by attachments to a nuclear matrix consisting of the nuclear lamina and an internal fibrogranular network made of nuclear matrix proteins and RNA (Nickerson, 2001). Ubiquitin regulates the cell cycle both by proteolytic and non-proteolytic mechanisms (Gilberto and Peter, 2017). Not only does ubiquitin impact all stages of DNA replication, particularly by protecting DNA from insults (García-Rodríguez et al., 2016; Moreno and Gambus, 2015), but it also drives cell cycle progression (e.g. cyclin ubiquitination and proteasomal degradation at metaphase/anaphase transition). Furthermore, ubiquitin plays a critical role in regulating the dynamics of nucleosomal chromatin structure (Gilberto and Peter, 2017) wherein nucleosome histones must be evicted from DNA and deposited in a semi-conservative manner onto new DNA strands, the gaps being filled with newly synthesized histones. Thus, to maintain genome integrity, nucleosome assembly during S-phase necessitates an adequate histone supply (Alabert and Groth, 2012), which is regulated by the processing factor stem-loop binding protein (SLBP). Interestingly, histone mRNA processing is activated by CRL4WDR23 through multi-monoubiquitination of SLBP (Brodersen et al., 2016). Cells lacking SLBP exhibit severe DNA replication defects. After S-phase, SLBP is rapidly degraded by SCF cyclin F complexes (Dankert et al., 2016), and this proteolytic degradation is critical for genome maintenance upon genotoxic stress. Thus, the non-proteolytic and proteolytic modes of regulation of SLBP by ubiquitin cooperate in space and time to restrict histone synthesis to S phase and thereby maintain genome stability. These mechanisms may also provide explanations for the misshaped nuclear structure, small sized nucleus and fragmented blastomere nucleus in Cas9 gRNA injected embryos in the current study. If so, *Uba52* gene knockout would reduce cellular ubiquitin content and cause a severe insult to DNA and cell functions, resulting in abnormal nuclear morphology and developmental arrest. With *Uba52* being a fusion gene, the UBA52 protein is formed by co-translation of full length 76-amino acid (AA) mono-ubiquitin linked through its C-terminus to the N-terminus of 52 AA ribosomal/ribonucleo-protein (RNP) CEP52 (Redman and Burris, 1996). The UBA52 as one of the ubiquitin-tail fusion RNPs is involved in the ribosomal biogenesis (Finley et al., 1989) and their relative abundance in cells correlates with the cellular content of assembled ribosomes (Redman and Burris, 1996). Therefore, it was very possible that ribosomal biogenesis was likely affected as well.

In previous studies, CRISPR/Cas9 gRNA was injected into zygote/1-cell stage porcine embryos to modify genes that are not essential for pre- or post-implantation development (Whitworth et al., 2017). However, *Uba52* is classified as a housekeeping gene (Ahn et al., 2008; Sadritdinova et al., 2014; Schoen et al., 2015) and is likely activated before major embryonic genome activation (Latham and Schultz, 2001; Østrup et al., 2013). Thus, the injection time window had to be moved to metaphase II stage oocyte, i.e. prior to fertilization and oocyte activation, to assure timely modification of the Uba52 prior to first embryo mitosis. High efficiency of such a gene modification indicates that the current method was efficient to interfere with development.

In conclusion, the present study demonstrated that the CRISPR/Cas9 of *Uba52* gene significantly reduced (and completely prevented in bi-allelic conformation) porcine blastocyst formation *in vitro,* decreasing UBA52 and proteasomal subunit protein content, and causing developmental arrest at 4-cell to 8-cell stage and blastomere nuclear deformation. We thus conclude that *Uba52* plays an essential role in the pre-implantation embryo development.

## MATERIALS AND METHODS

### Reagents and antibodies

All chemicals used in this study were purchased from Sigma Chemical Co. (St. Louis, USA) unless otherwise stated. Rabbit polyclonal antibody against the 20S proteasomal core subunits was purchased from Enzo Life Sciences Inc. (catalog #PW8155, Farmingdale, USA) and validated in previous studies by western blotting and immunocytochemistry (Zigo et al., 2018). Two anti-UBA52 antibodies were used in the present study. The UBA52 monoclonal antibody was from Abcam (catalog #ab109227, Cambridge, USA) and UBA52 polyclonal antibody from Thermo Fisher Scientific (catalog #PA5 23685, Rockford, USA). Affinity purified goat anti-rabbit IgG FITC secondary antibody was obtained from Invitrogen.

### Design of gRNAs to build specific CRISPRs

Five 17–20 bp guides were designed to target the sequence located adjacent to an S. pyogenes (Spy) protospacer adjacent motif (PAM) (Ran et al., 2015) within exon 1 of *Uba52* gene. The targets were selected by the following method. Repeat Masker (Smit and Green, 1996) (‘Pig’ repeat library) was used to identify any repetitive elements in the *Uba52* genomic sequence and these areas were not used as potential targets. Specificity of each potential guide was confirmed by searching for similar porcine sequences in GenBank (https://www.ncbi.nlm.nih.gov/genbank/). If guides and the adjacent PAM sequence had similarity to other areas of the genome, they were removed from subsequent analysis. In addition, structural analysis of the 20 bp guide with the CRISPR RNA (crRNA) and the trans-activating crRNA (tracrRNA) (Hsu et al., 2013) was evaluated for potential disruption of gRNA structure by mFold (http://unafold.rna.albany.edu). If potential guides were predicted to form an appropriate ‘handle’ to interact with Cas9 and were not predicted to form a tight hairpin that could potentially prevent interaction with the genome, they were added to the finalized list of potential guides. Five guides were chosen for the experiment based on the criteria listed above. The five guides and the PAM sequences (Fig. 3A) were: Guide 1, ATCTTTGTGAAGACCCTGA**CGG**; Guide 2, GATAAGGAGGGTGAGTT**GGG**; Guide 3, CCAACTCACCCTCCTTATCC**TGG**; Guide 4, ACATTCTCAATGGTATCACT**GGG** and Guide 5, TATCACTGGGCTGACCTCA**GGG**.

### *In vitro* transcription of single guide RNAs for the CRISPR/Cas9 system

Template guide DNA was first synthesized by Integrated DNA Technologies in the form of a gBlock. A T7 promoter sequence was added upstream of the guide for *in vitro* transcription. Each gBlock was diluted to final concentration 0.1 ng/μl and PCR amplified with a gBlock F primer (ACTGGCACCTATGCGGGACGAC) and a gBlock R primer (AAAAGCACCGACTCGGTGCCAC) with Q5 (New England Biolabs, Ipswich, MA) following standard protocol. PCR conditions consisted of an initial denaturation of 98°C for 1 min followed by 35 cycles of 98°C (10 s), 68°C (30 s) and 72°C (30 s). Each PCR amplified gBlock was purified by using a QIAGEN (Valencia, USA) PCR purification kit following standard protocol. Purified gBlock amplicons were used as template for *in vitro* transcription by standard protocol with the MEGAshortscript T7 transcription kit (Ambion; Thermo Fisher Scientific) followed by purification using the MEGAclear T7 clean-up kit (Ambion). Quality of the synthesized RNAs were visualized on a 2.0% RNA-free agarose gel and concentrations 260:280 ratios were determined via spectrophotometry. Polyadenylated *Cas9* mRNA containing 5-methylcytidine and pseudouridine modifications was used (TriLink Biotechnologies). Five gRNAs were mixed together then with *Cas9* mRNA and diluted in nuclease-free water at a final concentration of 20 and 20 ng/μl, respectively. Prepared RNA was divided into 5 μl aliquots and stored at −80°C until oocyte injection.

### Porcine oocyte collection and *in vitro* maturation

Detailed procedures for oocyte collection and *in vitro* maturation have been described previously (Abeydeera et al., 1998). Briefly, ovaries from pre-pubertal gilts were collected at a local slaughterhouse and transported to the laboratory in a warm box (25–30°C). Cumulus-oocyte complexes (COCs) were aspirated from antral follicles (3–6 mm in diameter). Oocytes with uniform ooplasm and compact cumulus were collected, and washed three times in HEPES-buffered Tyrode lactate (TL-HEPES) medium containing 0.1% (w/v) polyvinyl alcohol (PVA) and one time with the maturation medium (Abeydeera et al., 1998). Batches of fifty COCs were transferred to 500 μl of the maturation medium that had been covered with mineral oil in a 4-well plate (Nunc) and equilibrated at 38.5°C, 5% CO_2_ in air. Oocyte maturation medium was tissue culture medium (TCM) 199 (Mediatech, Manassas, USA) supplemented with 0.1% PVA, 3.05 mM D-glucose, 0.91 mM sodium pyruvate, 0.57 mM cysteine, 0.5 μg/ml LH, 0.5 μg/ml FSH, 10 ng/ml EGF, 10% porcine follicular fluid, 75 μg/ml penicillin G and 50 μg/ml streptomycin. After 40 h *in vitro* maturation, cumulus cells were removed with 0.1% hyaluronidase in TL-HEPES-PVA medium and the oocytes were washed three times and transferred into TL-HEPES-PVA medium (pH 7.4) for microinjections.

### Cytoplasmic injection of metaphase II oocytes with CRISPR/Cas9+gRNAs

Microinjection was performed on the heated stage of a Zeiss Axiovert-35 inverted microscope (Zeiss, Jena, Germany) fitted with Eppendorf micromanipulators and Femtojet 5247 injector (Eppendorf, Hauppauge, USA). Glass micropipettes with an outer diameter of 1.0 mm and an inner diameter of 0.78 mm were pulled to a fine point of <1.0 μm (Sutter Instrument, Navato, USA). The mixture of CRISPR RNA (crRNA, 100 ng/µl) and 20 ng/µl of gRNAs was microinjected into cytoplasm of oocyte (designated as Uba52gRNA group). crRNA (100 ng/μl) without gRNAs was injected as a control. Surviving oocytes were washed three times in fertilization medium and used for IVF.

### *In vitro* fertilization, embryo culture and assessment of development

Injected oocytes were placed into 100 μl drops of a modified Tris-buffered medium (mTBM) containing caffeine and BSA, covered with mineral oil, which had been equilibrated for 48 h at 38.5°C in 5% CO_2_ in air as described (Mao et al., 2012). The dishes were kept in a CO_2_ incubator until spermatozoa were added for fertilization. Sperm-rich ejaculate fraction from a boar of known fertility was collected weekly on Tuesdays and used for IVF on Wednesdays and Thursdays. The semen was checked for motility (minimum 80%) right after collection, and kept at room temperature (24°C). For IVF, 4 ml of semen was centrifuged at 600 ***g*** for 10 min to remove the seminal plasma. The supernatant was discarded, and the sperm pellet was re-suspended at room temperature in the BTS extender (Minitube, Delavan, USA) after sperm concentration was adjusted to 1×10^8^ cells/ml. On fertilization day, semen was diluted in mTBM. The processed semen was added in to oocyte-containing fertilization droplets at a final sperm concentration of 1×10^4^ cells/ml. Oocytes were co-incubated with spermatozoa for 5–6 h at 38.5°C, 5% CO_2_ in air, at which time point the putative zygotes were washed three times and transferred to four-well plates containing 500 ml of zygote culture medium (Mao et al., 2012) for additional incubation at 38.5°C, in 5% CO_2_ in air. The number of embryos cleaved on day 2, the number of 2-, 4- and 8-cell stage embryos on day 4, and the number of blastocysts formed on day 7 after fertilization (IVF day=0), were recorded under a stereomicroscope. Only embryos with blastomeres of equal size were counted to determine the numbers of 2-, 4- and 8-cell stage embryos.

### PCR screening for insertions and deletions

PCR assay was designed to assess the presence of *Uba52* gene edits including insertions and deletions (INDELs) in the resulting embryos with an amplicon size of 305 bp. Sense and antisense primer sequences were: Uba52F, AGGCATAGGGCTGGCAGTCT and Uba52R, TCCGTCCACACAGGACAGCA.

INDELs were determined by PCR amplification of the *UBA52* gene in the Exon 1 region flanking the projected cutting site introduced by the CRISPR/Cas9 system. PCR conditions of the INDELs assay consisted of an initial denaturation of 94°C for 1 min followed by 37 cycles of 94°C (30 s), 52°C (30 s) and 68°C (15 s) finishing with a final extension at 72°C for 2 min 30 s. Resulting amplicons were then visualized by electrophoresis using a 4% agarose gel.

### TOPO cloning and DNA sequencing

The resulting PCR products were Sanger DNA sequenced at the University of Missouri DNA Core facility. PCR amplicons from each embryo were TOPO cloned using the TOPO TA kit (Thermo Fisher Scientific) by following standard protocol. Clones were propagated on Luria–Bertani (LB) agarose plates containing 50 μg/ml kanamycin and resistant recombinants were selected. Plasmids containing the *Uba52* amplicon were identified by EcoRI digestion, and subsequent DNA agarose gel electrophoresis. Plasmids that contained the *Uba52* amplicon were DNA sequenced at the University of Missouri DNA core by using the Uba52F oligonucleotide. Sequences were aligned to the wild-type *Uba52* gene and INDELS were examined.

### Immunofluorescence

A standard immunofluorescence procedure for labeling embryos against Uba52 (Sutovsky et al., 2005) was performed as follows. Immediately after embryo collection, zona pellucida was removed by a short, 5 s incubation in acidic PBS (pH 1.79). Embryos were fixed in 2% (v/v) formaldehyde for 40 min at room temperature, washed, permeabilized in phosphate-buffered saline (PBS) containing 0.1% (v/v) Triton X-100, and blocked for 25 min in 0.1 M PBS containing 5% normal goat serum (NGS) and 0.1% Triton X-100. Samples were incubated overnight at 4°C with primary antibody diluted at 1:200 in 0.1 M PBS containing 1% NGS and 0.1% Triton X-100. On the following day, after a wash in PBS, the primary antibodies were detected by a mixture of goat anti-rabbit IgG-FITC diluted 1:100 and DNA stain DAPI (4,6-diamidino-2-phenylindole, 2.5 μg/ml), incubated at room temperature for 40 min. Negative controls were obtained by the replacement of primary antibody with normal rabbit serum at the immunoglobulin concentration matching that of the relevant specific antibody. Embryos were mounted on slides using Vectashield anti-fade mounting medium (Vector Laboratories Inc., Burlingame, USA), and observed with a 40× and 60× infinity-corrected objectives using a Nikon Eclipse 800 microscope (Nikon Instruments, Melville, USA). Images of individual embryos were acquired using a high-resolution Cool Snap CCD camera (Roper Scientific, Tucson, USA) and MetaMorph software (v7.1, Universal Imaging, Downington, USA) with a fixed setting for all images. Images were cropped, sized and arranged into panels using Photoshop CC version 2017 (Adobe Systems). Quantification of intensity of labeling in the equatorial plane of entire embryos was performed using Image Studio Lite software (v5.2, LI-COR Biotechnology, Lincoln, USA). The circumference of the embryo was selected and the mean intensity obtained using the analysis tool of Image Studio. Background intensity was obtained from the area surrounding the embryo using the same technique and the value subtracted from embryo intensity.

### Number of nuclei in embryos

The number of nuclei in the fixed embryos was determined after counter staining with DAPI as described above. Live embryos used for DNA preparation and PCR assays were stained with 20 mM Hoechst 33342 dye (2′-[4-ethoxyphenyl]-5-[4-methyl-1-piperazinyl]-2,5′-bi-1H-benzimidazole trihydrochloride trihydrate) and mounted on slides in culture medium. The number of nuclei was used as an estimate of the number of cells in an embryo.

### SDS/PAGE and western blotting-densitometry

Western blotting method described previously (Miles et al., 2013; Zigo et al., 2018) was used to determine the UBA52 protein levels in embryos with a little modification. After the zona pellucida was removed, embryos were washed three times in PBS and boiled with loading buffer [50 mM TRIS (pH 6.8), 150 mM NaCl, 2% SDS, 20% glycerol, 5% β-mercaptoethanol, 0.02% bromophenol blue]. Gel electrophoresis was performed on 4-20% gradient gels (PAGEr Gels; Cambrex Bio Science, Rockland, USA), followed by transfer to PVDF membranes (Millipore, Bedford, USA) using an Owl wet transfer system (Fisher Scientific) at a constant 50 V for 4 h. The membranes were incubated sequentially with 10% non-fat milk for 1 h at room temperature, primary antibody (#ab109227, Abcam) at 1:1000 dilution overnight at 4°C, and HRP-conjugated goat anti-rabbit antibody (1:10,000 dilution) for 40 min at room temperature. The membranes were reacted with chemiluminescent substrate (Luminata Crescendo Western HRP Substrate; Millipore). Blots were screened with ChemiDoc Touch Imaging System (Bio-Rad) to visualize the protein bands and analyzed by Image Lab Touch Software (Bio-Rad). Unless otherwise specified, procedures were carried out at room temperature. Residual gels and membranes after chemiluminescence detection were stained with Coomassie Brilliant Blue (CBB) R-250 (both Thermo Scientific) for protein normalization (Zigo et al., 2018). The UBA52 band intensity was normalized based on both the band density of Coomassie staining of residual gel and the number of embryos used preferentially to normalization on actin/tubulin, the quantities of which are affected by cell number and developmental competence. Negative control was obtained by the replacement of primary antibody with a non-immune rabbit serum.

### Statistical analysis

Dependent variables were analyzed for normality by using the Wilk–Shapiro test (SAS, 2014). Data for the dependent variables, percentage of zygotes cleaved on day 2 (cleavage rate), and percentage of zygotes forming an apparent blastocysts (blastocyst formation rate) were arcsine-transformed. Data on per cent of zygotes cleaved on day 2, per cent of blastocysts formed on day 7, number of nuclei in day 7 blastocyst and relative intensity of immunolabeling of UBA52 and proteasome were analyzed by analysis of variance using the PROC GLM procedure of SAS (SAS, 2014). The proportion of 2-, 4- and 8-cell stage embryos on day 4 of embryo culture, as a function determination of embryonic developmental potential between the Uba52 gRNA and control, was analyzed by FREQ procedure of SAS with X^2^ as an option (SAS, 2014). For all variables, treatments were fixed effects and replicate was considered a random effect. A value of *P*<0.05 was considered statistically significant. In the results, the least-squares means and the standard errors of means are presented.
